# Clinicopathological Features of Intrathoracic Liposarcoma—A Systematic Review with an Illustrative Case

**DOI:** 10.3390/jcm11247353

**Published:** 2022-12-10

**Authors:** Kajetan Kiełbowski, Nikola Ruszel, Seweryn Adam Skrzyniarz, Małgorzata Edyta Wojtyś, Rafał Becht, Konrad Ptaszyński, Darko Gajić, Janusz Wójcik

**Affiliations:** 1 Department of Thoracic Surgery and Transplantation, Pomeranian Medical University in Szczecin, Alfreda Sokołowskiego 11, 70-891 Szczecin, Poland; 2 Department of Internal Medicine and Hypertension with Subdepartment of Cardiology, Independent Public Provincial Hospital in Szczecin, Alfreda Sokołowskiego 11, 70-891 Szczecin, Poland; 3 Dietrich-Bonhoeffer-Klinikum, 17036 Neubrandenburg, Germany; 4 Department of Clinical Oncology, Chemotherapy and Cancer Immunotherapy, Pomeranian Medical University in Szczecin, Unii Lubelskiej 1, 71-252 Szczecin, Poland; 5 Department of Pathology, Faculty of Medicine, Collegium Medicum, University of Warmia and Mazury in Olsztyn, 10-561 Olsztyn, Poland

**Keywords:** thoracic surgery, liposarcoma, intrathoracic liposarcoma, well-differentiated liposarcoma

## Abstract

Background: Liposarcoma (LPS) is one of the most common soft-tissue sarcomas. However, intrathoracic LPS is rare, as only 1% of all LPS cases are found in the thorax. Methods: A systematic literature review through PubMed and Embase databases was performed. Only eligible case reports and case series reporting intrathoracic LPS in adult patients were included. Kaplan–Meier curves were calculated to evaluate the survival rate of included patients based on the histological subtype of LPS. Results: 123 studies reporting 197 patients were included. We added a case of a 69-year-old female patient with recurrent giant intrathoracic LPS. The primary tumor measured 15.1cm × 22.9 cm × 21.9 cm and weighed 3100 g. Six months later, the patient was admitted to the hospital with another intrathoracic tumor measuring 9.5 cm × 9 cm× 1.4 cm. The immunohistochemical studies showed expression of murine double minute 2 (MDM2) antigen in both primary and recurrent tumor cells. Conclusions: Dyspnea, chest pain, and cough were the most common symptoms reported in included studies. Overall, the 5-year survival rate was 62%. The highest survival was observed in well-differentiated LPS patients (80%) and the lowest in myxoid LPS (31%).

## 1. Introduction

Liposarcoma (LPS) is one of the most frequent soft-tissue malignancies, accounting for 20% of all cases. LPS originates from abnormal differentiation of adipocytes or lipoblasts and usually develops in patients between the 5th and 7th decade of life [1]. On the other hand, less than 1% of mediastinal tumors are LPSs [2]. Discovery of those tumors is frequently incidental, and the clinical course is asymptomatic until the lesion obtains significant measures and begins to displace and compress surrounding structures. The aim of this article is to provide valuable insight into clinicopathological features of intrathoracic LPS with an illustrative case and a systematic review of the literature.

## 2. Case Report

**Figure 1:**
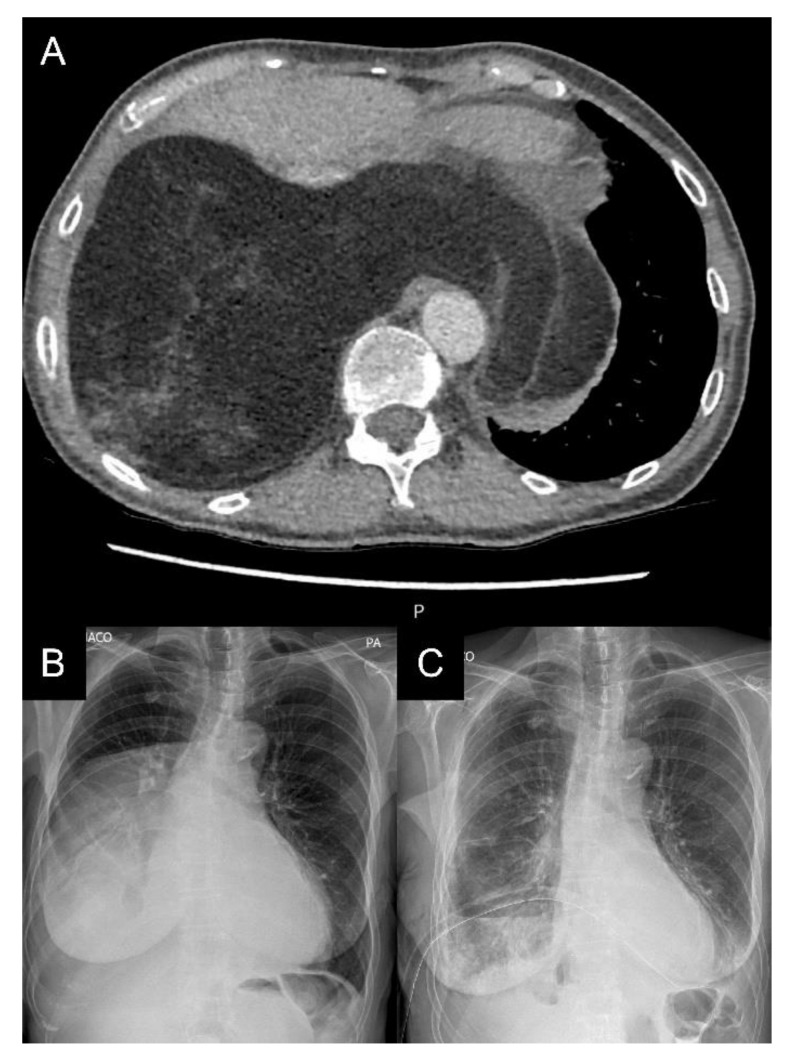
Radiological imaging of the patient during the first surgery. (**A**): CT with a massive lesion in the right thoracic cavity, 15.1 cm × 22.9 cm × 21.9 cm in size. (**B**,**C**): Pre- and postoperative chest X-rays of the patient.

**Figure 2:**
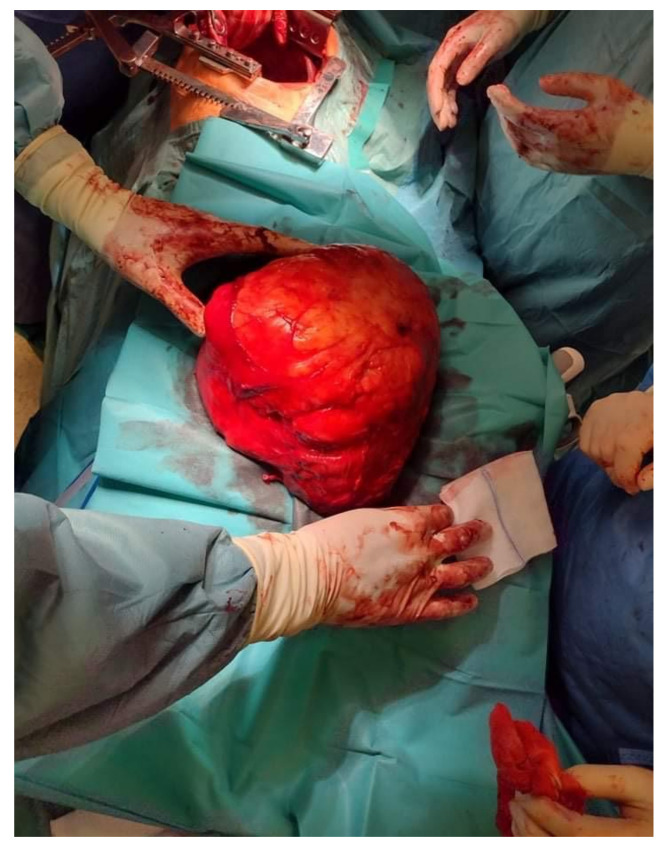
Intraoperative picture of the giant intrathoracic liposarcoma.

**Figure 3:**
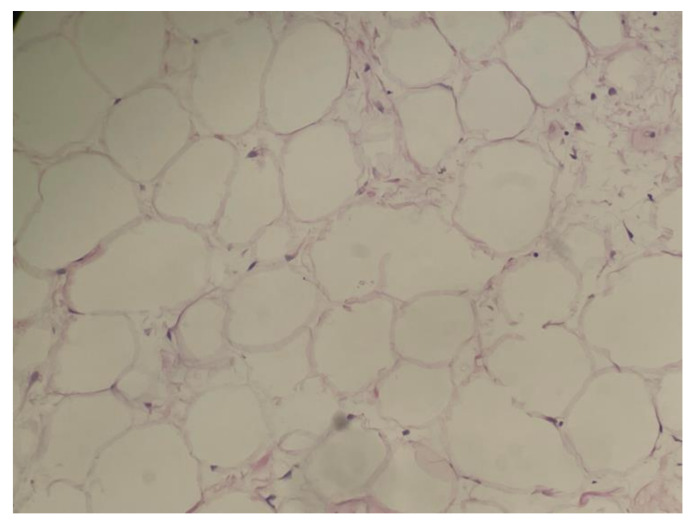
Atypical Lipomatous Tumor (ALT)/Well-Differentiated Liposarcoma (WDL). Lipoma-like histological appearance, with most of the tumor showing mature adipose tissue histology. Only rare, scattered spindle stromal cells with hyperchromatic nuclei were observed. (H&E; 400×).

We report on a 69-year-old female patient admitted to the Department of Cardiology due to gradually increasing dyspnea and weakness for the past two years. Throughout this period, the patient remained under regular cardiological control. Her medical history included gastrectomy due to cancer 30 years prior, thyroidectomy due to a goiter 20 years earlier, hysterectomy due to fibroids, cholecystectomy, and lipoma resection of the neck region. The patient quit smoking 30 years prior to admission. Before that, the patient had smoked for 20 years. In addition, she suffered from coronary artery disease, atrial fibrillation, and arterial hypertension. The patient was in the process of qualifying for cardiac resynchronization therapy (CRT). However, a chest X-ray showed an abnormal mass in the right thoracic cavity. During hospitalization, computed tomography (CT) confirmed a massive lesion in the right thoracic cavity, 15.1 cm × 22.9 cm × 21.9 cm in size, with a 5 cm strand passing to the left side between the heart and descending aorta. The tumor had a heterogeneous adipose tissue density and contained soft tissue lesions and calcifications (Figure 1). The lesion was compressing the lower and middle lobes of the right lung and pushing the ipsilateral right main bronchus and carina upwards. It was compressing the esophagus and displacing the heart forward. A complete representation of a tumor suggested the presence of an adipose lesion with an origin in the pleura. Thoracic wall involvement was not observed. The CRT therapy was postponed, and the patient was transferred to the Department of Thoracic Surgery for further investigation and treatment. Upon admission, the patient was in good physical condition. The laboratory blood tests were within normal ranges. The echocardiography showed an ejection fraction (EF) of 40% without segmental contractility disorders of the heart walls. According to a bronchofiberoscopy, which is a typical preoperative examination, the bilateral lobar bronchi were unobstructed. An endobronchial ultrasound revealed the tumor in the region of the intermediate bronchus. Transbronchial needle aspiration biopsy (TBNA) did not confirm neoplastic infiltration. The patient qualified for surgical treatment in February 2022. A muscle-sparing thoracotomy through the right sixth intercostal space revealed a well-defined, encapsulated tumor measuring 25 cm× 17 cm× 12 cm. At closure, two chest drains were implemented and intercostal nerve block was performed. The weight of the mass was 3100 g. The tumor was resected en bloc (Figure 2). On cross-section, it was solid with necrotic areas. Histopathological examination confirmed well-differentiated liposarcoma with sparse, scattered lipoblasts. (Figure 3). The postoperative period was uneventful. Daily drainage in the 1st, 2nd, 3rd, and 4th postoperative days were 50 mL, 50 mL, 200 mL, and 120 mL, respectively. Since there was no air leak, the first and the second drains were removed on the 1st and 5th day after surgery, respectively. The patient was discharged from the hospital on the 8th postoperative day with the recommendation of further oncological control close to the residence.

**Figure 4:**
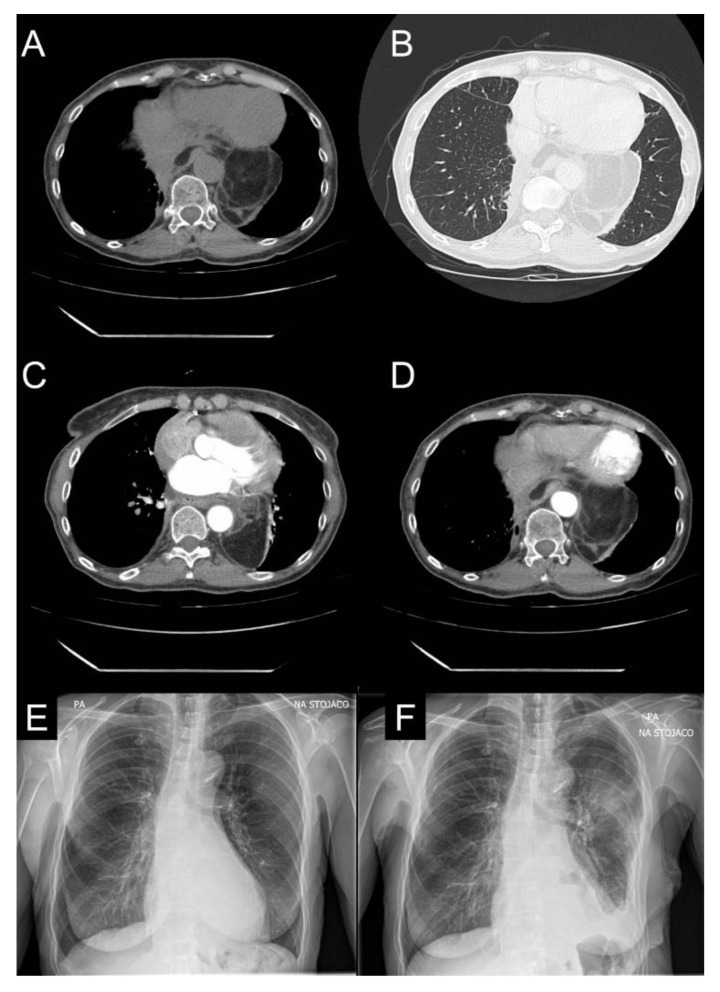
Radiological imaging of the patient during the second surgery. (**A**–**D**): CT images from August 2022 with a massive lesion in the posterior mediastinum. (**E**,**F**): Pre- and postoperative chest X-rays of the patient from 21 September 2022 and 29 September 2022, respectively.

In August 2022, a control chest CT revealed a recurrent tumor measuring 9.5 cm × 9 cm × 1.4 cm in the posterior mediastinum (Figure 4). Therefore, in September 2022, the patient was admitted to the Department of Thoracic Surgery. The EF on admission was 55%. On the 21st of September, a muscle-sparing thoracotomy through the left sixth intercostal space revealed a soft tissue tumor surrounding the inferior part of the thoracic aorta. Moreover, the lesion showed adhesions with the pericardium and left lower lobe. In addition, a small lesion was identified in the pulmonary parenchyma. Therefore, the mediastinal mass was resected and a segmentectomy of the left lower lobe was performed. On macroscopic examination, the mediastinal mass consisted of two separate tumors measuring 55 mm × 48 mm × 20 mm and 40 mm × 35 mm × 10 mm; the pulmonary lesion measured 35 mm × 22 mm × 12 mm. The histopathology of the mediastinal tumors showed adipose lesions with foci of lipoblast concentration, features consistent with WDLPS and areas showing low-grade dedifferentiation (DDLPS). The character of the pulmonary lesion was benign. The postoperative course was uneventful and the average daily drainage was 200 mL with no air leak. On the 7th postoperative day, 50 mL was drained and the chest drain was removed. The patient was discharged from the hospital in good physical condition. The immunohistochemical studies showed expression of murine double minute 2 (MDM2) antigen in several cells, both primary and recurrent tumor cells.

## 3. Materials and Methods

**Figure 5:**
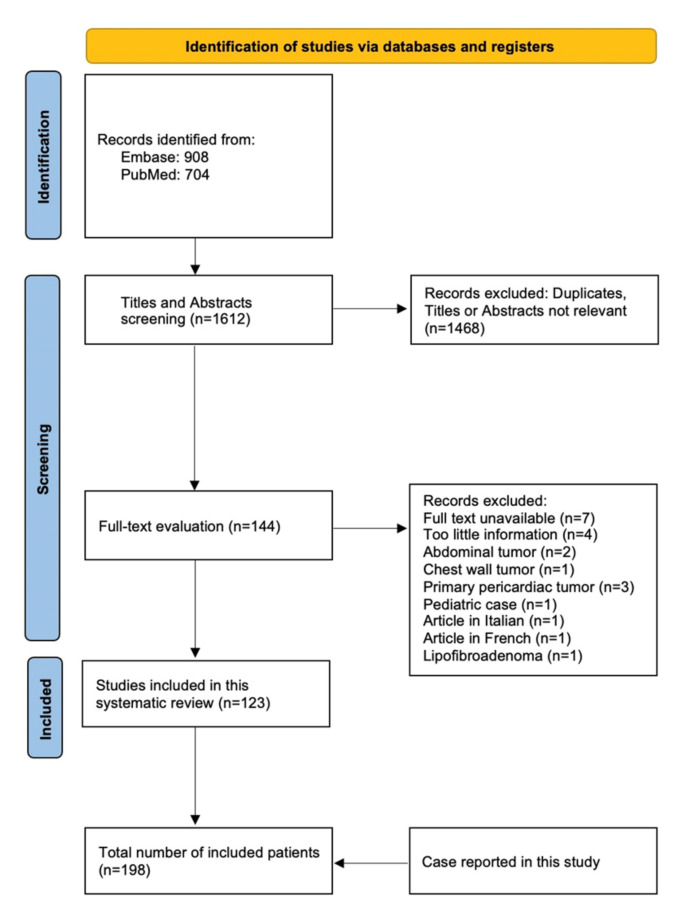
Flow diagram of the selection process.

The article follows CARE guidelines for case reports and the Preferred Reporting Items for Systematic Reviews and Meta-Analysis (PRISMA) [3,4]. Informed consent for the publication was obtained from the patient. A thorough literature search through the PubMed and Embase databases was performed using the keywords: ‘thoracic liposarcoma’ OR ‘intrathoracic liposarcoma’ OR ‘giant thoracic liposarcoma’ OR ‘mediastinal liposarcoma’ OR ‘pleural liposarcoma’ OR ‘thoracic liposarcoma case report’. The primary results consisted of 908 articles in Embase and 704 in PubMed. To be included, a study needed to be a case report or case series of intrathoracic liposarcoma written in English. Cases of chest wall sarcomas describing pediatric patients and studies published prior to 1996 were excluded. Primary esophageal and pericardial LPS were not included. A search strategy is presented in a flow diagram (Figure 5). Two independent reviewers performed a systematic search, while three reviewers manually extracted data. In case of uncertainty, the issue was resolved through the consensus of three reviewers. Kaplan–Meier curves were calculated to present the survival of LPS patients. Raw data can be found in the Supplementary Materials.

## 4. Results

A total of 123 case reports and case series with 198 patients were included in this systematic review. The clinicopathological characteristics of included patients are presented in Table 1. During the full-text evaluation, 21 studies were excluded. Thirty-two (26%) articles were published by authors from Japan, 17 (14%) from the USA, 16 (13%) from China, and 11 (9%) from India. The mean age of included patients was 55 years. A total of 78% of patients were 45 years old or older. Liposarcoma originated from mediastinal tissues, pleura, or lungs in 75%, 17%, and 7%, respectively. In two patients, liposarcoma was found in the supraclavicular fossa. Symptoms were reported in 148 patients. Twenty-four (16%) were asymptomatic with tumors found incidentally. Dyspnea was the most common symptom appearing in 50% of included patients, followed by chest pain (29%), cough (26%), and dysphagia (13%). Weight loss and fatigue were less common (7% and 6%, respectively). Duration of symptoms varied from several days to 3 years. Physical examinations often revealed decreased breath sounds and dullness on percussion. Treatment was reported in 195 patients. The vast majority (96%) of patients underwent surgical excision. The surgical approach depends on tumor location, size, and infiltration. It may include posterolateral thoracotomy, median sternotomy, hemiclamshell, clamshell, or video-assisted thoracoscopic surgery (VATS) together with thymectomy, lung parenchyma resection, or partial resection of the pericardium. Surgery may be accompanied by extracorporeal membrane oxygenation in the case of hemodynamic instability in patients with mediastinal tumors [5]. Adjuvant or neoadjuvant radio- and chemotherapy was introduced in 34 and 20 patients, respectively. The most frequently observed tumor size ranged from 10 to 20 cm and appeared in 41% of patients. Tumors smaller than 10 cm occurred in 23%, while the largest LPS (between 20 and 30 cm; larger than 30 cm) were observed in 21% and 15% of included patients, respectively. The largest mean tumor size was observed in WDLPS and measured 20 cm. The mean DDLPS size measured 16 cm, while both MLPS and PLPS were 14 cm. Nevertheless, the largest reported tumor in this review measured 50 cm and was a DDLPS [6]. Tumor weight was reported in 54 patients; 37% of patients presented with LPS heavier than 3000 g and 33% with lighter than 1000 g. Histological subtype was reported in 191 patients. Well-differentiated liposarcoma (WDLPS) was the most common histology (44%), followed by dedifferentiated (DDLPS, 21%), myxoid (MLPS, 19%), and pleomorphic (PLPS, 9%). Macroscopically, LPS usually presents as an encapsulated, yellow, and lobulated mass. Histologically, WDLPS comprises mature adipocytes admixed with stromal cells showing hyperchromatic nuclei. In MLPS, the myxoid matrix is typically present, as well as various numbers of round cells. DDLPS and PLPS are histologically characterized by numerous pleomorphic and bizarre, atypical cells. Table 2 describes pathological characteristics of intrathoracic LPSs from selected studies that reported detailed immunohistochemistry data. Typical LPS markers included MDM2, cyclin-dependent kinase 4 (CDK4), and p16. MDM2 was reported in 29 patients, of whom 28 were WDLPS and DDLPS. Follow-up data were reported in 163 patients. Mean and median follow-up period of included patients were 29 and 15 months, respectively. Overall, the 5-year survival rate was 62%. The highest survival was observed in WDLPS patients (80%) and the lowest in MLPS (31%). Recurrent disease was observed in 37% of patients and developed from 6 to 96 months postoperatively.

## 5. Discussion

Liposarcoma is a mesenchymal tumor first reported by Virchow in 1860 [7]. In 2013, the WHO divided LPS into four histological subtypes: well-differentiated (WDLPS), dedifferentiated (DDLPS), myxoid (MLPS), and pleomorphic (PLPS). Updated classification from 2020 added an extremely rare myxoid pleomorphic liposarcoma due to improvements in immunodiagnostic methods. LPS has an aggressive clinical course with significant recurrence and metastatic rates. WDLPS further subdivides into inflammatory, sclerosing, and lipoma-like variants [8,9]. WDLPS is considered to be the most frequent and develops in approximately half of all patients with LPS, which is consistent with our review. However, extremities are a typical location for these neoplasms [10]. Primary intrathoracic sarcomas are rare; most of the cases with histological features of sarcoma are metastatic. Nevertheless, they may arise from various chest tissues, including all compartments of the mediastinum, pleura, pulmonary artery, or lung, which are exceptionally uncommon [11,12].

### 5.1. Clinical Course

The clinical course of LPS patients mostly depends on tumor location and size. In this review, 16% of patients were asymptomatic, with tumors found incidentally. In time, the tumor starts to compress adjacent structures and patients become symptomatic. Dyspnea was the most common complaint (50%), followed by chest pain (29%). Our patient also reported dyspnea on admission and CT revealed compression of the right middle and lower lobes. A similar clinical presentation was observed by Baheti et al., who reviewed patients with intrathoracic synovial sarcoma. A total of 21% of patients were asymptomatic, while 49% and 19% of complaints represented chest pain and dyspnea, respectively [13]. According to a study by Xiao et al., 44% of retroperitoneal LPS patients were asymptomatic; 32% and 28% of patients presented abdominal distortion or pain, respectively [14].

### 5.2. Radiological Imaging

The most frequent diagnostic procedures in this systematic review included CT, MRI, PET, and biopsy. A CT scan of LPS showed heterogeneous mass with nodules, fibrotic, and adipose tissues [15]. On the other hand, lipomas are usually homogenous on imaging, but it may be difficult to differentiate lipoma and WDLPS [16]. In the case of WDLPS, CT demonstrates fat density by low Hounsfield units (HU); higher density is observed in the case of DDLPS [17]. Therefore, a lower CT value of LPS indicates more dedifferentiation [18]. MRI is a useful diagnostic tool that can evaluate the invasion of surrounding structures and characterize tissue components [19]. In the presented case, it was decided that postponing the procedure for an MRI scan could have detrimental effects due to the present symptoms and tumor size. In the case of large mediastinal LPS, several authors did not include preoperative MRI scan in the diagnosis process either [18,20,21].

### 5.3. Treatment

As demonstrated in this review, surgical resection is the most common treatment strategy in intrathoracic LPS (188 patients (96%)). Complete resection is one of the most significant factors in LPS treatment. Leaving even a small fragment of the tumor or its capsule worsens survival and increases the risk of recurrence. Proper surgical access determines treatment success. Minor lesions can be successfully removed using the VATS technique. Open surgeries are usually performed in larger lesions. Typically, a posterolateral or anterolateral thoracotomy is used, depending on the location of the lesion and the operator’s preference. The anterior mediastinal lesions are more demanding and may involve large vessels. However, in certain clinical situations, a more extensive incision, which provides a better exposure, is required. The literature describes the removal of such lesions by bilateral anterior thoracotomy, median sternotomy, or clamshell access. Yet another example is tumors infiltrating the posterior mediastinum. Taki et al. described a demanding case that required simultaneous esophageal resection [22]. Large tumors causing pressure on the cardiac muscle, as in the case described above, may generate cardiac arrhythmias, which should disappear after the lesion is removed. Additionally, filling a large part of the pleura causes pressure on the lung. In people whose lesions develop over a long period of time, even after the removal of the lesion, one of the significant problems may be the difficulty in lung expansion after the surgery.

Furthermore, additional RTH was reported in 35 patients. A similar strategy is performed in retroperitoneal LPS [23]. There is no consensus regarding the use of radiotherapy in LPS. According to Lee et al., LPS patients with histology other than WDLPS experienced less recurrence after combining RTH and surgery. However, statistical significance was not met (*p* = 0.087) [24]. Findings of a recent phase 3 clinical trial (STRASS) revealed that preoperative RTH might benefit patients with LPS, and this issue needs to be further explored [25]. MLPS was found to be highly sensitive to radiation treatment, which translated into the use of preoperative radiotherapy in this type of liposarcoma [26]. Chemotherapy was reported in 20 patients and included ifosfamide, adriamycin, lipozonid, doxorubicin, docetaxel, or pazopanib. Pazopanib is not approved for the treatment of LPS, although preclinical data indicate its activity in DDLPS [27]. Current evidence remains controversial regarding the use of chemotherapy in LPS patients. Clinical trials did not demonstrate prolonged overall survival and suggested that efficacy might depend on the histological subtype of sarcoma [28]. MLPS and PLPS are more sensitive to chemotherapy than WDLPS and DDLPS [29]. Eribulin is a new anticancer agent which destabilizes microtubules and inhibits spindle formation. It was approved by the FDA in 2010 to treat advanced breast cancer and in 2017 for the treatment of inoperable liposarcoma [30]. A phase 3 clinical trial confirmed that eribulin given to patients with advanced, metastatic, or recurrent LPS or leiomyosarcoma increases overall survival compared to dacarbazine [31]. The use of eribulin has been reported in a patient with intrathoracic LPS after intolerance of doxorubicin [7]. Another promising treatment agent in the treatment of LPS is trabectedin. It is a marine-derivative that interacts with DNA repair proteins. It has been demonstrated that trabectedin, as compared to dacarbazine, decreases the risk of disease progression or death in patients with LPS or leiomyosarcoma [32]. In a retrospective analysis of studies on the effectiveness of trabectedin, it was shown to be more effective in patients with specific genetic alterations, such as translocation responsible for the development of the neoplastic process, mainly in MLPS [33]. In a multicenter analysis of trabectedin in advanced MLPS, which included 51 patients, the results involved a complete response in 3.9% and a partial response in 47.1% of patients. A total of 51% of patients achieved an objective response and the median 6-month progression-free survival was 88% [34]. Additionally, the molecular landscape of LPS suggests that the patients may benefit from immunotherapy. For instance, Chae et al. revealed that 31.5% and 51.3% of WDLPS and DDLPS, respectively, expressed programmed death-ligand-1 (PDL-1) [35]. Therefore, clinical efficacy of immune checkpoint inhibitors (ICIs), such as nivolumab and ipilimumab, have been evaluated and the response may differ according to histology. DDLPS patients are considered to be potential candidates for the treatment with ICIs. Multiple clinical trials will further evaluate the treatment with other immunotherapies, such as CAR T cells or cytokine administration, among others [36].

### 5.4. Pathology

**Figure 6:**
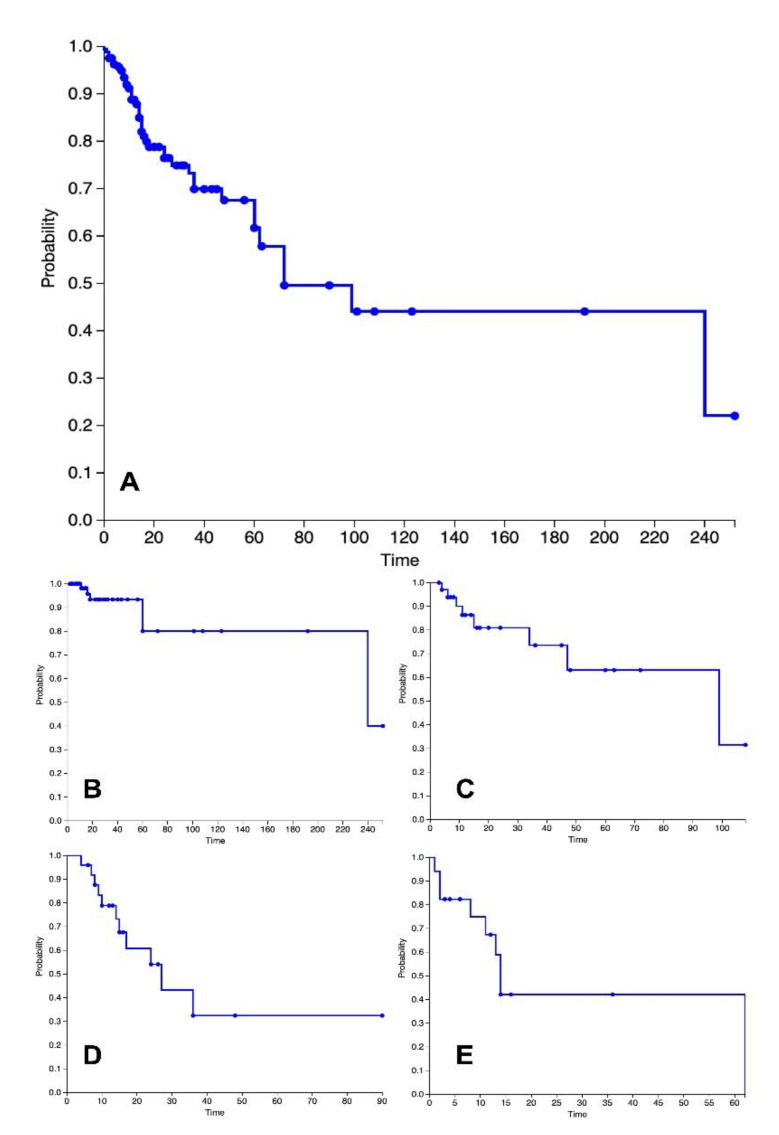
Kaplan–Meier survival curves based on histological subtypes: (**A**): all subtypes; (**B**): Well-differentiated liposarcoma; (**C**): Dedifferentiated liposarcoma; (**D**): Myxoid liposarcoma; (**E**): Pleomorphic liposarcoma.

Due to its uncommon location and many potential histological presentations, there is a broad differential diagnosis, including thymolipoma mesothelioma, teratoma, leiomyosarcoma, or fibrosarcoma. Additionally, there are various tumors composed of adipose tissue with different prognosis and management (e.g., lipoma, WDLPS, DDLPS, hibernoma). Therefore, immunohistochemical and molecular methods have been studied extensively in LPS. WDLPS and DDLPS develop due to amplification of the 12q13-15 chromosome segment. As a result, in both of those tumors, amplification of MDM2 (suppression of p53) and CDK4 (cell cycle regulator) oncogenes can be found by immunohistochemistry or in situ hybridization (ISH) [36]. Since the sensitivity of large atypical cells, which are distinctive for WDLPS, is not high, the MDM2 testing facilitates the differentiation of WDLPS from lipoma preoperatively [37]. MDM2 is also being evaluated as a potential target for future treatment. An in vivo study of the MDM2 inhibitor showed significant anti-tumor activity in DDLPS mice [38]. It has been demonstrated that CDK4 is overexpressed in 90% of DDLPS, making it a potential target currently evaluated in clinical trials [39]. An immunohistochemical triad of MDM2, CDK4, and p16 is important to differentiate WDLPS and DDLPS from other adipocytic tumors [40]. On the contrary, MLPS does not express amplification of MDM2 or CDK4. Instead, MLPS is associated with DNA-inducible transcript 3 (DDIT3), t (12;16) (q13;p11), which can be detected using RT-PCR or fluorescent in situ hybridization (FISH) [41]. PLPS shows complex chromosomal aberrations, and no marker has been identified to improve diagnostic procedures [42]. WDLPS does not usually give metastasis, but local recurrence is observed depending on the original tumor location. For instance, the retroperitoneal tumor has a greater risk of recurrence than LPS in the extremities [43]. Chae et al. reviewed clinical courses of 332 LPS tumors in the abdomen/pelvis, extremities, thorax, and head-neck regions. Overall, 40.7% of patients suffered from recurrence and in most cases, it was a local recurrence. 62% and 50% of tumors in the abdomen/pelvis and thorax recurred, respectively. On the contrary, only 15% of LPS located in the extremities relapsed. Furthermore, DDLPS and PLPS were found to have higher recurrence rates than WDLPS and MLPS [35]. In the case of retroperitoneal LPS, it has been determined that recurrence frequently develops between 6 months to 2 years after surgery [44]; in the presented patient, recurrence was detected 6 months after the initial surgery. In this review, metastases developed in 19 patients. Involved organs included lungs, liver, adrenal glands, or bones. Overall, the 5-year survival rate was 62%. The highest survival was observed in the WDLPS group (80%). Survival rates of DDLPS, MLPS, and PLPS were 64%, 31%, and 41%, respectively (Figure 6). Other studies reporting experience with retroperitoneal LPS patients had similar results (5-year survival, 65%; 4-year survival, 70%) [23,45]. As demonstrated by Vos et al., the location of LPS strongly affects the outcomes. The best prognosis was observed in patients with testicular LPS (5-year survival 93%). The 5-year survival for the common group of retroperitoneal and intrathoracic LPS was 62.2% [46].

**Table 2:** Immunohistochemical and genetic characteristics of selected case reports/case series describing intrathoracic liposarcoma.

Reference	Histology	S100	Vimentin	Desmin	SMA	CD31	CD34	CD36	MDM2	CDK4	p16	HHF-35	EMA	CK	BCL2	CD99
[47]	WDLPS	1	1				1									
[48]	WDLPS	1							1							
[49]	WDLPS								1							
[49]	WDLPS	0		0					1							
[49]	WDLPS	1		1					1							
[50]	WDLPS	0	1	1	1		1			1			0		0	0
[51]	WDLPS	1		1	0		1		1							
[52]	WDLPS								0	0						
[53]	WDLPS								1	1	1					
[21]	WDLPS	0	1	0	0		1									
[54]	WDLPS	1		1	1											
[55]	WDLPS								1							
[56]	WDLPS								1							
[57]	WDLPS								1	1						
[58]	WDLPS						1		1							
[59]	WDLPS								1	1	1					
[60]	WDLPS								1							
[61]	WDLPS			1	1		1									
[61]	WDLPS			1			1									
[62]	WDLPS								1							
[63]	DDLPS			1	1		1					1				
[64]	DDLPS	1		0	0		0		1	1		0				
[65]	DDLPS	0		0	0											
[66]	DDLPS								1	1						
[67]	DDLPS	0		0	1		0					0	0		0	
[68]	DDLPS								1	1						
[49]	DDLPS	0		0	0				1							
[49]	DDLPS	0		1	1				1							
[69]	DDLPS								1	1						
[70]	DDLPS								1	1						
[71]	DDLPS		1				1		1							1
[6]	DDLPS	1	1											0		
[72]	DDLPS								1	1						
[73]	DDLPS								1	1	1					
[74]	DDLPS	1/0	1	0	0				1					0		
[75]	DDLPS								1							
[76]	DDLPS								1							
[77]	DDLPS	1	1	1	0		1									
[78]	DDLPS	0	1						1	1				1		1
[79]	MLPS	1	1										0	0		
[80]	MLPS	1	1					0					0			
[81]	MLPS	1	1	0	0		1									
[82]	MLPS	1			0											
[82]	MLPS	1			0											
[49]	MLPS	1		0					0							
[49]	MLPS	1		0					0							
[49]	MLPS	1		0					0							
[83]	MLPS	0	1	0	1	0	0							0		
[84]	PLPS	1		0	0	0	0							0		
[85]	PLPS	1	1	1									0			

### 5.5. Limitations

This systematic review cannot be considered without certain limitations. Firstly, articles in languages other than English and without full-text access were excluded. Therefore, we could have missed some case reports of high relevance. Secondly, selection bias should be acknowledged. Furthermore, only a certain number of studies reported detailed information regarding radiological or pathological findings and follow-up. Intrathoracic LPS is a rare disease mostly reported in case reports and case series. More prospective studies with large sample sizes are necessary to understand clinicopathological features fully.

## 6. Conclusions

To conclude, intrathoracic LPS is a rare neoplasm. Symptoms, if present, are usually due to compression of the surrounding organs. Due to the uncommon location, heterogeneous features in imaging, and multiple possible histological presentations, differential diagnosis is broad. Recent advances in pathology facilitate a precise preoperative diagnosis, which is essential for proper patient management. As demonstrated in this review, only a complete surgical resection offers the best possible outcomes. There are no guidelines regarding additional use of radio- and chemotherapy and this issue should be further examined. Unresectable LPS might be treated with eribulin, while further treatment agents are under constant examination with promising results. LPS patients require a long-term follow-up period, as recurrence may develop years postoperatively.

## Figures and Tables

**Table 1:** Clinicopathological characteristics of included patients. SD—standard deviation; MDM2—murine double minute 2; CDK4—cyclin-dependent kinase 4; WDLPS—well-differentiated liposarcoma; DDLPS—dedifferentiated liposarcoma; MLPS—myxoid liposarcoma; PLPS—pleomorphic liposarcoma.

Characteristics	n (%)
Age Mean (SD)	55 (15)
Age ≥ 45	155 (78%)
Age < 45	43 (22%)
Male	120 (61%)
Female	78 (39%)
Tumor origin:	
Mediastinum	149 (75%)
Pleura	35 (17%)
Lung	13 (7%)
Supraclavicular fossa	2 (1%)
Symptoms	
Asymptomatic	24 (16%)
Chest pain	43 (29%)
Dyspnea	74 (50%)
Cough	39 (26%)
Weight loss	10 (7%)
Dysphagia	19 (13%)
Fatigue	9 (6%)
Features on radiological imaging	
Homogeneous	7
Heterogeneous	28
PET SUVmax mean (SD)	4.3 (2.7)
Surgical treatment	188 (96%)
Non-surgical treatment	7 (4%)
Recurrent disease	53 (37%)
Radiotherapy	35
Chemotherapy	20
Tumor size (max diameter)	
<10 cm	35 (23%)
≥10 cm and <20 cm	64 (41%)
≥20 cm and <30 cm	33 (21%)
≥30 cm	22 (15%)
Well-differentiated mean (range)	20 cm (4.5–40)
Dedifferentiated mean (range)	16 cm (3.5–50)
Myxoid mean (range)	14 cm (4–29.3)
Pleomorphic mean (range)	14 cm (4.5–25)
Tumor weight	
<1000 g	18 (33%)
≥1000 g and <2000 g	10 (19%)
≥2000 g and <3000 g	6 (11%)
≥3000 g	20 (37%)
Histological subtype	
Well-differentiated	84 (44%)
Dedifferentiated	41 (21%)
Myxoid	37 (19%)
Pleomorphic	18 (9%)
Immunohistochemistry/gene amplification	
MDM2	29 (34%)
CDK4	13 (15%)
p16	4 (5%)
5-year survival rate	62%
5-year survival rate WDLPS	80%
5-year survival rate DDLPS	64%
5-year survival rate MLPS	31%
5-year survival rate PLPS	41%

## Data Availability

Not applicable.

## Supplementary Materials

The following supporting information can be downloaded at: https://www.mdpi.com/article/10.3390/jcm11247353/s1, Raw data of this systematic review.
